# Osteoarticular Infections in Pediatric Hospitals in Europe: A Prospective Cohort Study From the EUCLIDS Consortium

**DOI:** 10.3389/fped.2022.744182

**Published:** 2022-05-04

**Authors:** Andreas Trobisch, Nina A. Schweintzger, Daniela S. Kohlfürst, Manfred G. Sagmeister, Matthias Sperl, Andrea J. Grisold, Gebhard Feierl, Jethro A. Herberg, Enitan D. Carrol, Stephane C. Paulus, Marieke Emonts, Michiel van der Flier, Ronald de Groot, Miriam Cebey-López, Irene Rivero-Calle, Navin P. Boeddha, Paul-Michael Agapow, Fatou Secka, Suzanne T. Anderson, Uta Behrends, Uwe Wintergerst, Karl Reiter, Federico Martinon-Torres, Michael Levin, Werner Zenz, The EUCLIDS consortium

**Affiliations:** ^1^Department of Pediatrics and Adolescent Medicine, Division of General Pediatrics, Medical University of Graz, Graz, Austria; ^2^Division of Neonatology, Department of Pediatrics and Adolescent Medicine, Medical University of Graz, Graz, Austria; ^3^Research Group for Neonatal Infectious Diseases, Medical University of Graz, Graz, Austria; ^4^Department of Orthopedics and Traumatology, Pediatric Orthopedic Unit, Medical University of Graz, Graz, Austria; ^5^Diagnostic and Research Institute of Hygiene, Microbiology and Environmental Medicine, Medical University of Graz, Graz, Austria; ^6^Section of Pediatric Infectious Disease, Imperial College London, London, United Kingdom; ^7^Department of Clinical Infection Microbiology and Immunology, University of Liverpool Institute of Infection, Veterinary and Ecological Sciences, Liverpool, United Kingdom; ^8^Translational and Clinical Research Institute, Newcastle University, Newcastle upon Tyne, United Kingdom; ^9^National Institute for Health Research (NIHR) Newcastle Biomedical Research Centre Based at Newcastle upon Tyne Hospitals NHS Trust and Newcastle University, Newcastle upon Tyne, United Kingdom; ^10^Pediatric Infectious Diseases and Immunology Department, Newcastle upon Tyne Hospitals Foundation Trust, Great North Children's Hospital, Newcastle upon Tyne, United Kingdom; ^11^Department of Pediatrics, Division of Pediatric Infectious Diseases and Immunology and Laboratory of Infectious Diseases, Radboud Institute of Molecular Life Sciences, Nijmegen, Netherlands; ^12^Department of Pediatric Infectious Diseases and Immunology, Wilhelmina Children's Hospital University Medical Center Utrecht, Utrecht, Netherlands; ^13^Translational Pediatrics and Infectious Diseases Section- Pediatrics Department, Santiago de Compostela, Spain; ^14^Instituto de Investigación Sanitaria de Santiago (IDIS), Genetics- Vaccines- Infectious Diseases and Pediatrics Research Group (GENVIP), Santiago de Compostela, Spain; ^15^Department of Pediatrics, Erasmus MC-Sophia Children's Hospital, University Medical Center Rotterdam, Rotterdam, Netherlands; ^16^Medical Research Council Unit the Gambia, Banjul, Gambia; ^17^Department of Pediatrics and of Pediatric Surgery, Technische Universität München, Munich, Germany; ^18^Department of Pediatrics, Hospital St. Josef, Braunau, Austria; ^19^Department of Pediatric Intensive Care, University Children's Hospital at Dr. von Haunersche Kinderklinik, Ludwig-Maximilians-Universität München, Munich, Germany

**Keywords:** pediatric osteomyelitis, pediatric septic arthritis, Europe, EUCLIDS, *S. aureus*

## Abstract

**Background:**

Pediatric osteoarticular infections (POAIs) are serious diseases requiring early diagnosis and treatment.

**Methods:**

In this prospective multicenter cohort study, children with POAIs were selected from the European Union Childhood Life-threatening Infectious Diseases Study (EUCLIDS) database to analyze their demographic, clinical, and microbiological data.

**Results:**

A cohort of 380 patients with POAIs, 203 with osteomyelitis (OM), 158 with septic arthritis (SA), and 19 with both OM and SA, was analyzed. Thirty-five patients were admitted to the Pediatric Intensive Care Unit; out of these, six suffered from shock, one needed an amputation of the right foot and of four left toes, and two had skin transplantation. According to the Pediatric Overall Performance Score, 36 (10.5%) showed a mild overall disability, 3 (0.8%) a moderate, and 1 (0.2%) a severe overall disability at discharge. A causative organism was detected in 65% (247/380) of patients. *Staphylococcus aureus* (*S. aureus*) was identified in 57.1% (141/247) of microbiological confirmed cases, including 1 (0.7%) methicillin-resistant *S. aureus* (MRSA) and 6 (4.2%) Panton-Valentine leukocidin (PVL)-producing *S. aureus*, followed by Group A *Streptococcus* (18.2%) and *Kingella kingae* (8.9%). *K. kingae* and PVL production in *S. aureus* were less frequently reported than expected from the literature.

**Conclusion:**

POAIs are associated with a substantial morbidity in European children, with *S. aureus* being the major detected pathogen. In one-third of patients, no causative organism is identified. Our observations show an urgent need for the development of a vaccine against *S. aureus* and for the development of new microbiologic diagnostic guidelines for POAIs in European pediatric hospitals.

## Introduction

Pediatric osteoarticular infections (POAIs) have been reported to occur with an estimated annual incidence of 1–13 per 100,000 children in developed, and up to 200 per 100,000 children in developing countries (1).

Advances in medicine have led to reduced mortality; however, morbidity associated with POAIs is substantial. Early diagnosis and treatment is crucial, as delays may lead to a high rate of sequelae, such as longitudinal growth arrest, stiffness, angular deformity, chronic infection, or complications, such as septicemia, avascular necrosis, multi-organ failure, and death (2, 3). POAIs mainly occur as a consequence of hematogenous dissemination of microorganisms. In children suffering from osteomyelitis (OM), *Staphylococcus aureus* (*S. aureus*) is the most common causative pathogen, followed by Group A *Streptococcus* (GAS) (4, 5).

Using microbiological technologies based on nucleic acid detection, such as real-time polymerase chain reaction (PCR), *Kingella kingae* (*K. kingae*) has been increasingly identified in POAIs in the last decades, detected in up to 82% of children with septic arthritis (SA) under 5 years (6–8).

Community-acquired methicillin-resistant *S. aureus* (CA-MRSA) has increased among hospitalized children with POAIs in some countries, particularly in the United States (9–12), while the prevalence of MRSA remains low in European children with bone and joint infections (13, 14). Nevertheless, *S. aureus* producing the necrotizing toxin Panton-Valentine leukocidin (PVL) is increasing in the European pediatric population with incidence rates of up to 18.6% (15). This virulence factor is associated with increased severity and may lead to persisting inflammatory responses, regardless of methicillin resistance (15, 16).

The aims of the study were to describe the disease burden of POAIs and the microbiological detection rates of causative organisms in our prospective multicenter cohort recruited within the European Union Childhood Life-threatening Infectious Diseases Study (EUCLIDS). We analyzed demographic and clinical data, microbiology as well as patient outcome.

## Methods

### Study Design

This study is based on patients recruited in the EUCLIDS (www.euclids-project.eu), funded by the European Union's Seventh Framework Programme (FP7). EUCLIDS was a prospective, observational, multicenter study aiming to determine the genetic basis of life-threatening bacterial infections of childhood. The network, consisting of 194 hospitals in nine European countries and 2 hospitals in The Gambia, Africa, recruited patients from July 2012 to May 2017. Harmonized procedures for inclusion and exclusion criteria as well as clinical syndrome definitions are published elsewhere (17). The study protocol was approved by the Ethical Committee of the Medical University of Graz (24-116 ex 11/12) and by the local Ethical Committees from all participating centers. The study was conducted adherent to the Declaration of Helsinki principles and Good Clinical Practice guidelines. Written informed consent was obtained from parents or legal guardians for each child before study inclusion.

### Study Patients

Children and young adolescents from 1 month to 18 years of age admitted to hospital with sepsis (or suspected sepsis) and/or severe focal infection were eligible for EUCLIDS. Sepsis was defined as a systemic inflammatory response syndrome (SIRS) plus suspected infection according to Goldstein et al. (18). For this study, prospectively recruited patients with bone and joint infections were selected from the EUCLIDS database. Clinical definitions for POAIs can be found in the Supplementary File A. Fifty-two patients from the Swiss center were excluded due to divergent inclusion criteria, together with 27 patients from the non-European arm The Gambia. Two additional patients were excluded, one suffering from chronic recurrent multifocal osteomyelitis (CRMO) and one with insufficient clinical information. In line with other POAI studies, patients were subdivided into three groups of ages for data analysis: infants (≤ 3months), young children (>3–60 months), and older children (>60 months) (14, 19, 20).

### Data Collection

For the present study, the EUCLIDS database was queried for demographics, illness severity, therapies, blood count, microbiology, operations, and outcome at hospital discharge. Orthopedic operations were defined as an open surgery, including arthroscopy. Procedures such as needle puncture or drainage alone were not classified as orthopedic surgeries.

For a better overview, localizations affected by osteoarticular infections (OAIs) were grouped into regions. In OM, the skull region also includes the frontal sinuses and temporomandibular joint, the shoulder region includes clavicle and scapula, and the lower arm region includes radius and ulna. The hand region in SA only contains carpal/metacarpal joints, whereas in OM, it describes phalanges, wrist, and certain fingers. In SA, the pelvis describes only the sacroiliac joint, whereas in OM, it includes sacral, iliac, ischial, and pubic bone. The foot region in OM includes os calcalneum, os talus, joints distal to ankle joints, tarsal/metatarsal bone, metatarsophalangeal joints, and toes. In SA, it describes only subtalar joints.

Microbiological diagnosis was performed according to locally available microbiological investigations including bacterial culture from sterile sites (blood, cerebrospinal fluid, and invasive diagnostic samples), and from unsterile sites (throat and wound swabs).

Identification and resistance testing were performed according to standard procedures and, where available, these included molecular techniques such as PCR. Primers used for PCR diagnostics are exemplary as shown in Supplementary Table 1 and account for at least 60% (15/25) of all PCRs performed in our cohort (8, 21).

Bacteria that were rarely detected (in only one to a maximum of three patients) were classified into a group named “other bacteria” and consisted of *Actinomyces oris, Fusobacterium necrophorum*, Group B *Streptococcus, Mycobacterium kansasii, Mycobacterium tuberculosis, Staphylococcus epidermidis, Staphylococcus hominis, Haemophilus influenzae, Streptococcus milleri, Staphylococcus warneri*, and other unidentified *Streptococci*.

At discharge, patient outcomes were categorized according to the Pediatric Overall Performance Category (POPC) score (22), determined either by direct observation or by chart review (Supplementary Table 2).

### Statistics

Data are presented as medians with interquartile ranges (IQRs) or numbers (percentage). For readability, percentages in all tables were rounded. Characteristics were compared across disease and age groups, using Kruskal–Wallis test with Dunn's correction or Mann–Whitney test (continuous data) as well as Fisher's exact tests or Pearson's chi-squared test (categorical data), as appropriate. Statistical significance was set at 0.05. To reduce the likelihood of false-positive results caused by multiple testing, α was adjusted with Bonferroni correction. Nonsignificant analyses are not shown.

For all analyses, a two-sided *p* value < 0.05 was considered significant. Statistical analyses were conducted in R (version 3.5.1) (23) and with the GraphPad Prism Software Version 8 (GraphPad Software, La Jolla, CA, USA).

## Results

### POAIs Characteristics

**Figure 1:**
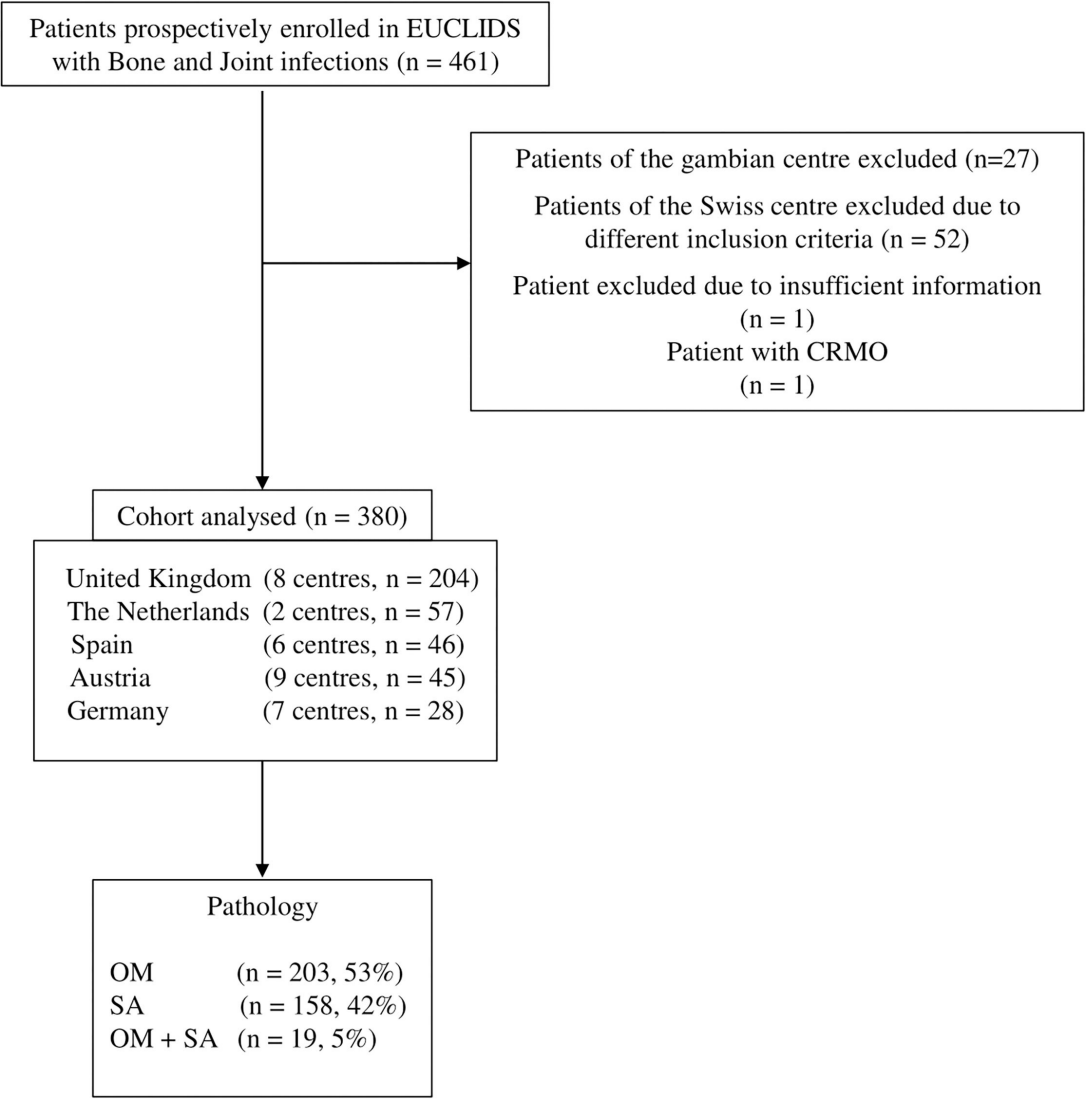
Flow diagram of patients with Bone and Joint infections eligible for this study.

We analyzed a cohort of 380 patients with POAIs, including 203 (53.4%) with OM, 158 (41.6%) with SA, and 19 (5%) with both OM and SA, recruited in five European countries (Figure 1). The median age at admission was 60 months (IQR, 17–122), and 162 (42.6%) of them were female (Table 1). Only 2.7% (10/380) of the whole cohort were infants (<3 months), whereas young and older children were almost evenly distributed [47.6% (181/380) and 49.7% (189/380), respectively] (Table 2). Young children were more often diagnosed with SA compared to OM [101/158 (63.9%) vs. 74/203 (36.4%), (*p* < 0.0001)] and OM + SA [101/158 (63.9%) vs. 6/19 (31.5%), (*p* = 0.011)]. Older children predominantly suffered from OM [OM vs. SA: 123/203 (60.5 %) vs. 54/158 (34.2%), (*p* < 0.0001)] (Table 2).

**Table 1:** Characteristics of patients with bone and joint infections.

	**All patients**	**OM**	**SA**	**OM + SA**	*P*-value^*^
No. of patients (%)	380	203 (53)	158 (42)	19 (5)	
Median age in months (IQR)	60 (17–122)	83^a^ (30–139)	29^a^ (14–82)	87 (19–149)	**<0.0001** ^a^
Gender; female (%)	162 (43)	86 (42)	70 (44)	6 (32)	0.571
Patients admitted to PICU (%)	35 (9)	21 (9)	10 (6)	4 (21)	0.068
Median days in PICU (IQR)	3 (2–7)	3 (2–7)	3 (2–6)	9 (4–10)	0.273
Median days of hospitalization (IQR)	9 (6–14)	10^a^ (6–15)	7^a, b^ (5–12)	18^b^ (10–26)	**0.0005**^a^ **0.0002**^b^
Days of IV^**^ antibiotic therapy (IQR)	8 (5–14)	11^a^ (7–16)	5^a^ (4–10)	14 (10–20)	**<0.0001** ^a^
Patients with systemic infections (%)	66 (17)	34 (17)	27 (12)	5 (26)	0.544
Patients with orthopedic operations (%)	213 (56)	82^a^ (40)	118^a^ (75)	13 (68)	**<0.0001** ^a^
No. of causative pathogens detected (%)	247 (65)	122 (60)	110 (70)	15 (79)	
*S.aureus* (%)	141 (57)	92^a^ (75)	39^a^ (36)	10 (67)	**<0.0001** ^a^
GAS (%)	45 (18)	18 (15)	24 (22)	3 (20)	0.350
*K.kingae* (%)	22 (9)	-^a^	22^a^ (20)	-	**<0.0001** ^a^
*S.pneumoniae* (%)	16 (6)	3 (2)	13 (12)	-	**0.011**
*N.meningitidis* (%)	5 (2)	1 (1)	4 (4)	-	0.411
*Salmonella* (%)	2 (1)	1 (1)	1 (1)	-	>0.999
Other bacteria (%)^***^	15 (6)	7 (6)	6 (5)	2 (13)	>0.999

* *P-values were calculated by comparing the OM-, SA- and OM + SA groups with each other. Kruskal-Wallis or Mann-Whitney test (continuous data) and Fisher's Exact tests (categorical data) were used, as appropriate. α was adjusted with Bonferroni correction*.

** *IV, intravenous*.

*** *Other bacteria reported as causative (n)*.

*Gr. B Streptococcus (n = 3), F. necrophorum (n = 1), S.epidermidis (n = 3), S. hominis (n = 1), S. warneri (n = 1), H. influenza (n = 1), A. oris (n = 1), S. milleri (n = 1), M. tuberculosis (n = 1), M. kansasii (n = 1), other unidentified Streptococci (n = 1)*.

a, b *Medians or total numbers on the same row are significantly different (P ≤ 0.05) when compared between groups*.

*Bold values indicate statistically significant (p-value < 0.05)*.

**Table 2:** Characteristics of patients with bone and joint infections subgrouped according to age into infants (≤ 3 months), young children (>3–60 months), older children (>60 months).

	**OM**			**SA**			**OM** **+** **SA**			***P*** -value^*^		
	**Infants**	**Young** **children**	**Older** **children**	**Infants**	**Young** **children**	**Older** **children**	**Infants**	**Young** **children**	**Older** **children**	**Infant** **groups**	**Young children** **groups**	**Older children** **groups**
Nr. of patients (%)	6 (3)	74^a^ (36)	123^c^ (61)	3 (2)	101^a, b^ (64)	54^c^ (34)	1 (5)	6^b^ (32)	12 (63)	0.44	**<0.0001**^a^ **0.011**^b^	**<0.0001** ^c^
Median age in months (IQR)	2 (1–2)	23 (13–39)	124 (89–153)	1 (1–1.5)	17 (12–30)	117 (80–144)	1.6	18 (15–24)	116 (87–151)	0.12	0.28	0.14
Females (%)	5 (83)	28 (38)	53 (43)	3 (100)	45 (45)	22 (41)	0	4 (67)	2 (17)	0.26	0.32	0.21
Patients with systemic infections (%)	0	9 (12)	25 (20)	0	14 (14)	13 (24)	0	1 (17)	4 (33)	-	0.78	0.45
Patients admitted to PICU (%)	1 (17)	7 (9)	13 (9)	0	6 (6)	4 (7)	0	0	4 (33)	>0.99	0.62	0.051
Median days in PICU (IQR)	7	2 (1–6)	3 (2–8)	0	3 (3–7)	3 (1–5)	0	0	9 (4–10)	-	0.41	0.39
Median days of hospitalization (IQR)	13 (9–33)	9 (5–14)	11 (7–17)	7 (4–10)	7 (5–11)	10 (6–15)	6	18 (11–22)	21 (10–31)	0.36	0.030	0.031
Days of IV antibiotic therapy (IQR)	8 (6–21)	9^a^ (5–14)	12 (9–20)	8 (5–14)	5^a^ (4–6)	10 (5–14)	-	14	15 (10–20)	0.80	**0.0008** ^a^	0.21
*S.aureus*	3	25^a^	64	-	10^a^	29	-	2	8	>0.999	**0.0003** ^a^	0.45
GAS	-	6	12	-	15	9	-	-	3	**-**	0.35	0.14
*K.kingae*	-	-	-	-	22	-	-	-	-	-	**<0.0001**	-
*S. pneumoniae*	-	2	1	-	10	3	-	-	-	-	0.16	0.13
*N.meningitidis*	-	1	-	-	3	1	-	-	-	-	0.682	0.339
*Salmonella*	-	-	1	-	1	-	-	-	-	-	>0.999	>0.999
Other bacteria	1	2	4	2	4	1	-	2	-	0.458	0.053	>0.999

* *P-values were calculated by comparing the OM-, SA- and OM + SA groups with each other. Kruskal-Wallis or Mann-Whitney test (continuous data) and Fisher's Exact tests (categorical data) were used, as appropriate. α was adjusted by Bonferroni correction*.

a, b, c *Medians or total numbers on the same row are significantly different (P ≤ 0.05) when compared between groups*.

*Bold values indicate statistically significant (p-value < 0.05)*.

In total, the number of patients diagnosed with a systemic infection was 66 of which 19 (28.7%) were admitted to the pediatric intensive care unit (PICU) (Table 1). Two patients suffered from septic shock and four from toxic shock syndrome. No patient died, one needed an amputation of the right foot and four left toes, and two received skin transplantation. No infant (<3 months) suffered from systemic infection (Table 2).

**Figure 2:**
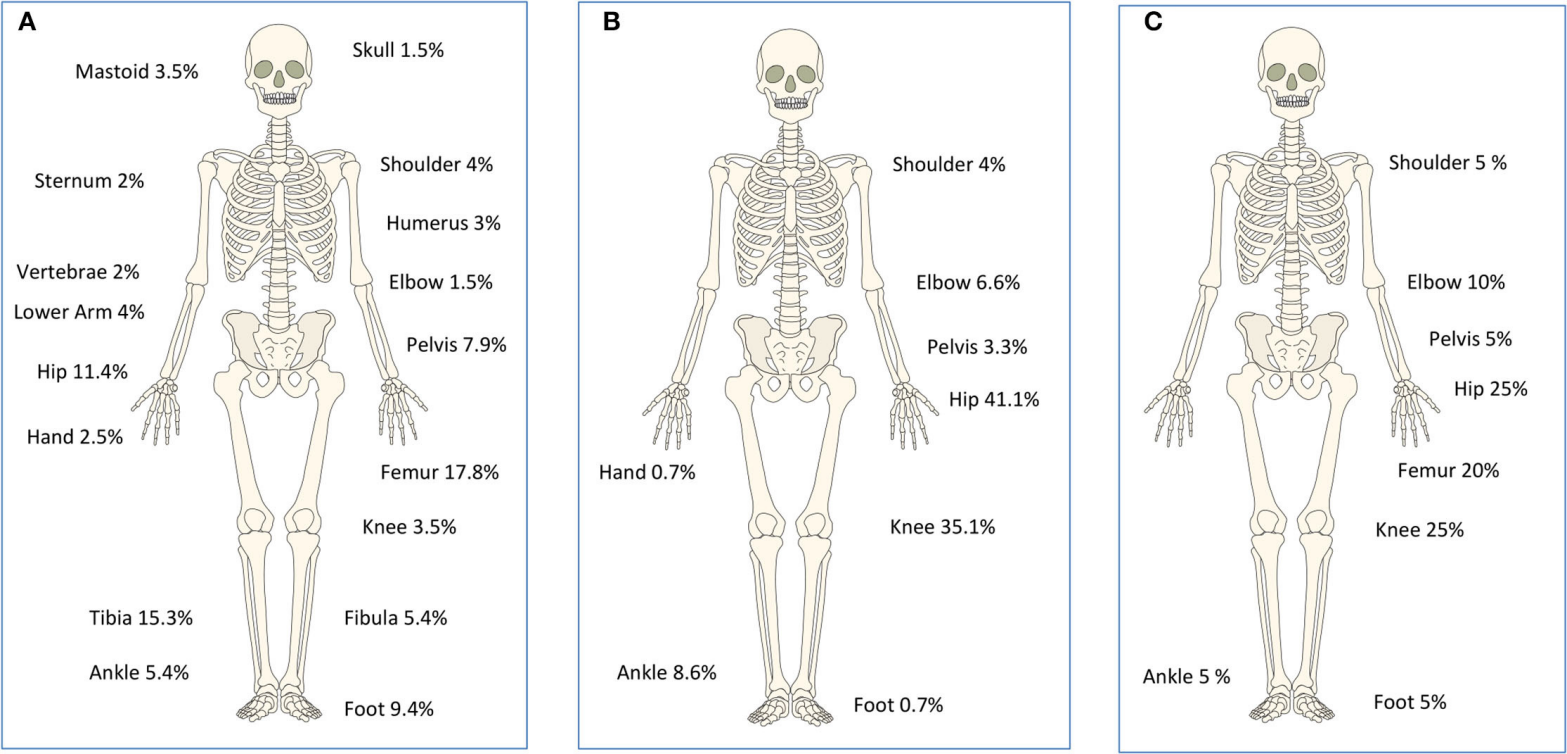
Anatomical locations affected in OM **(A)** SA **(B)** and OM + SA **(C)**. Cases with unknown locations: OM (*n* = 16), SA (*n* = 9) and OM + SA (*n* = 3). Cases with multifocal locations: OM (*n* = 9), SA (*n* = 3). This work “human bones” is a derivative of “Human bones.svg” taken from https://klexikon.zum.de/wiki/Datei:Human_bones.svg by Kazukiokumura used under CC by Andreas Trobisch.

Osteomyelitis mainly affected the femur [36/202 (17.8%)] and tibia [31/202 (15.3%)]. Patients with SA predominantly showed infections of the hip [62/151 (41.1%)] and the knees [53/151 (35.1%)] (Figure 2). Multifocal bone or joint infection sites were identified in 12 patients (9 with OM and 3 with SA). Thirty-one patients suffered from chronic OM (defined as beginning of symptoms >1 month before admission).

### Treatment and Outcome

Compared to patients with SA, patients with OM received longer intravenous antibiotic therapies (median of 11 days vs. 5, *p* < 0.0001) and had longer hospital stays (median of 10 days vs. 7, *p* = 0.0005).

Fifty-six percent (213/380) of patients needed orthopedic surgery. Patients with SA were more often submitted to operations than patients with OM [118/158 (74.6%) vs. 82/203 (40.3%), (*p* < 0.0001)] (Table 1) and showed a reduced mobility at discharge [SA vs. OM: 36/58 (62.1%) vs. 53/125 (42.4%), (*p* = 0.01)].

According to the Pediatric Overall Performance (POPC) Score (Supplementary Table 1), 301 of 340 (88.5%) patients with POAIs showed a good overall performance at discharge, 36 (10.5 %) a mild overall disability, 3 (0.8%) a moderate, and 1 (0.2%) a severe overall disability. More young children (3–60 months) with OM + SA showed a POPC score of two compared to the same age group with OM [OM + SA vs. OM: 3/5 (60%) vs. 6/64 (9.3%), (*p* = 0.013)] or SA [OM + SA vs. SA: 3/5 (60%) vs. 2/97 (2.1%), (*p* = 0.0006)].

### Pathogens

A causative organism was detected in 247/380 (65%) of patients. *S. aureus* was identified in 141/247 (57.1%) of all microbiological confirmed cases, followed by GAS in 45 (18.2%), *K. kingae* in 22 (8.9%), and *S. pneumoniae* in 16 (6.5%) cases (Table 1).

*Staphylococcus aureus* was more frequently detected in patients with OM compared to patients with SA [92/122 (75.4%) vs. 39/110 (35.4%), (*p* < 0.0001)] (Table 1).

**Figure 3:**
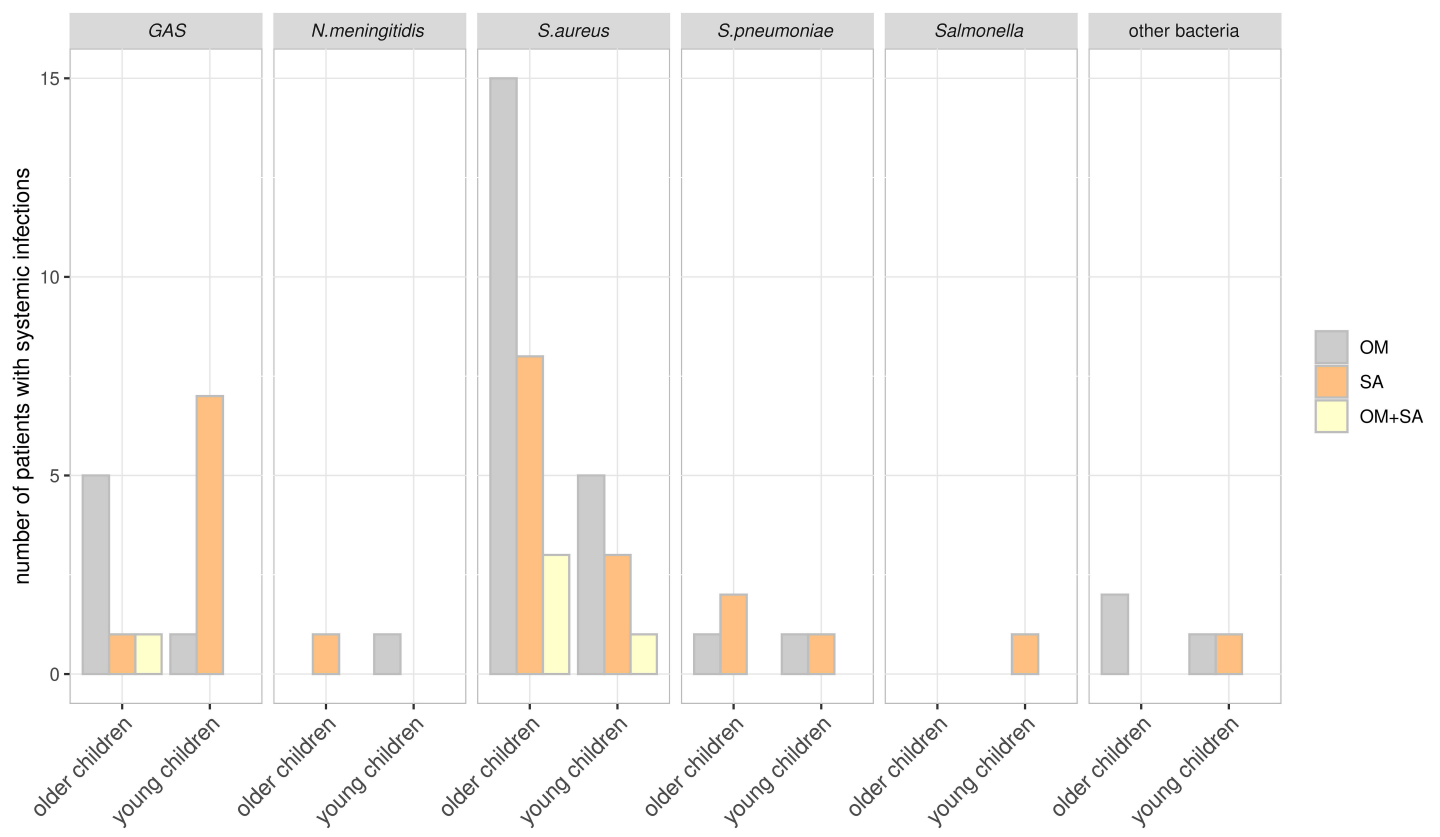
Patients with systemic infections and pathogens detected. Patients were subgrouped according to age at hospital admission. Young children: > 3–60 months; older children >60 months. Other bacteria: *F. necrophorum, H. influenzae, S. milleri* and *M. tuberculosis*.

In 66 cases with systemic infections, *S. aureus* was found in 35 (53%) patients, with 4 of them suffering from toxic shock syndrome (Figure 3). One patient was identified with MRSA, 6 with a PVL- positive *S. aureus*, and one with a toxic-shock-syndrome-toxin 1 (TSST-1) producing *S. aureus*.

Group A *Streptococcus* was found in 15/66 (22.7%) of patients with systemic infections and with high predominance in young children with systemic infections and SA [7/15 (56.6%)] (Figure 3 and Table 2).

*Kingella kingae* was only detected in young children with focal SA [22/101 (21.7%), (*p* < 0.0001)] (Table 2). *S. pneumoniae* was more frequently identified in patients with SA compared to patients with OM [13/110 (11.8%) vs. 3/122 (2.4%), (*p* = 0.011)] (Table 1). No differences in the number of identified pathogens were found between the age groups.

**Figure 4:**
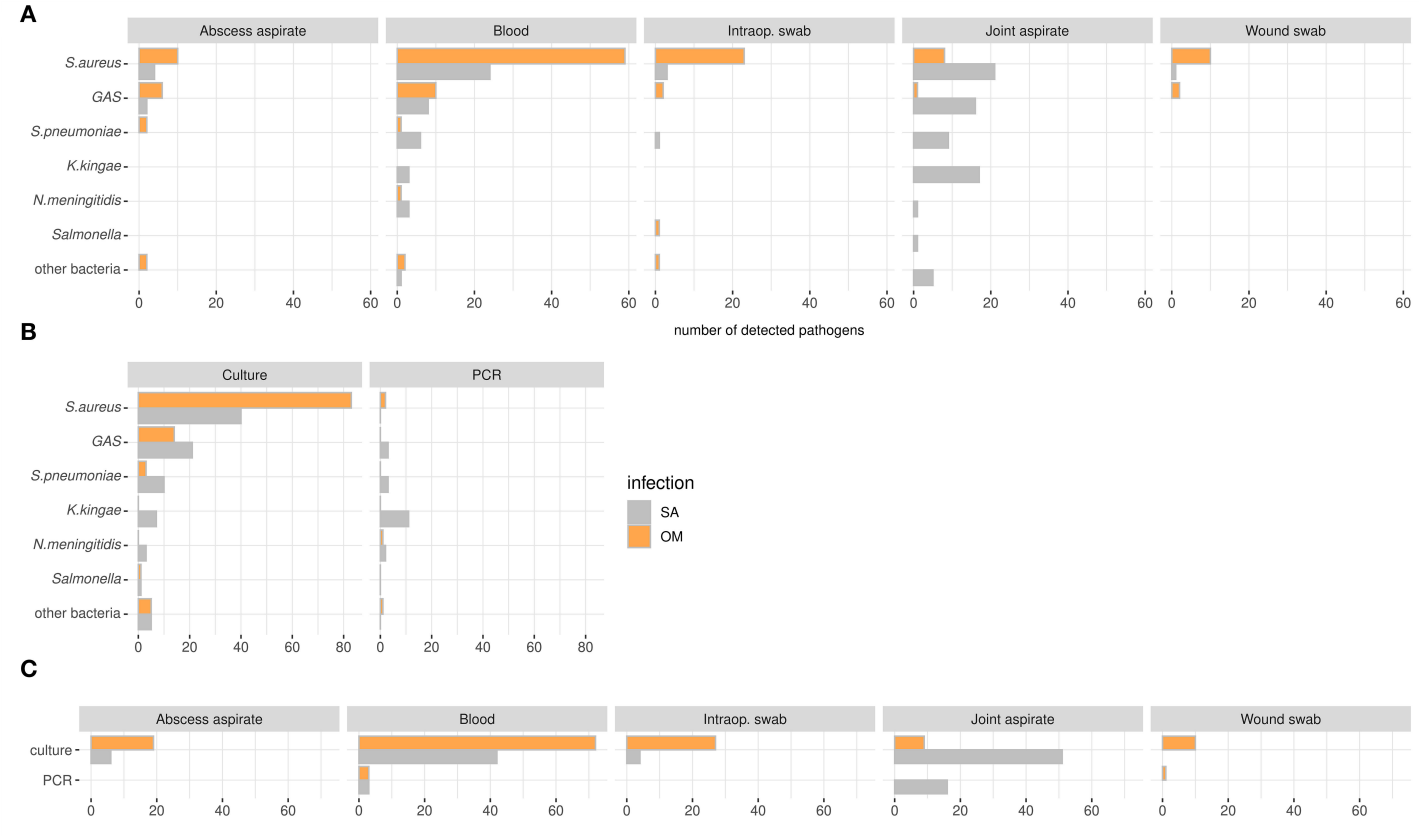
Pathogens displayed according to detection media **(A)** and -methods **(B)** and the methods according to biological samples **(C)** in the OAIs cohort. **(A)** Biological samples that are not shown (n): bone biopsies (*n* = 4), cerebrospinal fluid (*n* = 1), endotracheal tube aspirate (*n* = 2), and nose/throat swab (*n* = 2). **(B)** and **(C)** samples with unknown methods (*n*): 23. Patients with OM + SA are not shown.

Blood was the major source of causative pathogen detection in OM [73/141 (60%)], while this was the case for joint aspirate [70/126 (55.5%)] in SA (Figure 4A). The methods used for the identification of organisms were mainly culturing techniques in OM and SA [106/110 (96.3%) and 87/106 (82%)], followed by PCRs in SA [19/106 (17.9%)] (Figure 4B). *K. kingae* and GAS were predominantly identified in joint aspirate of patients with SA, while the main method of detection for *K. kingae* was PCR and culture for GAS (Figure 4B). Culture yielded positive results from blood more often in patients with OM [72/114 (63.2%)] than in patients with SA [42/114 (36.8%)]; the latter showed culture-positive pathogen detection more often when taken from joint aspirate [51/60 (85%) vs. OM 9/60 (15%)]. PCR-positive pathogen detection from joint aspirate was only seen in patients with SA (Figure 4C).

Past medical history showed five patients with sickle cell disease [3/203 (1.5%) with OM and 2/158 (1.3%) with SA]. In three of these cases, no pathogen was found, whereas in one case *S. pneumoniae* was identified and in one case *S. aureus*.

### Neutrophils and C-Reactive Protein

**Figure 5:**
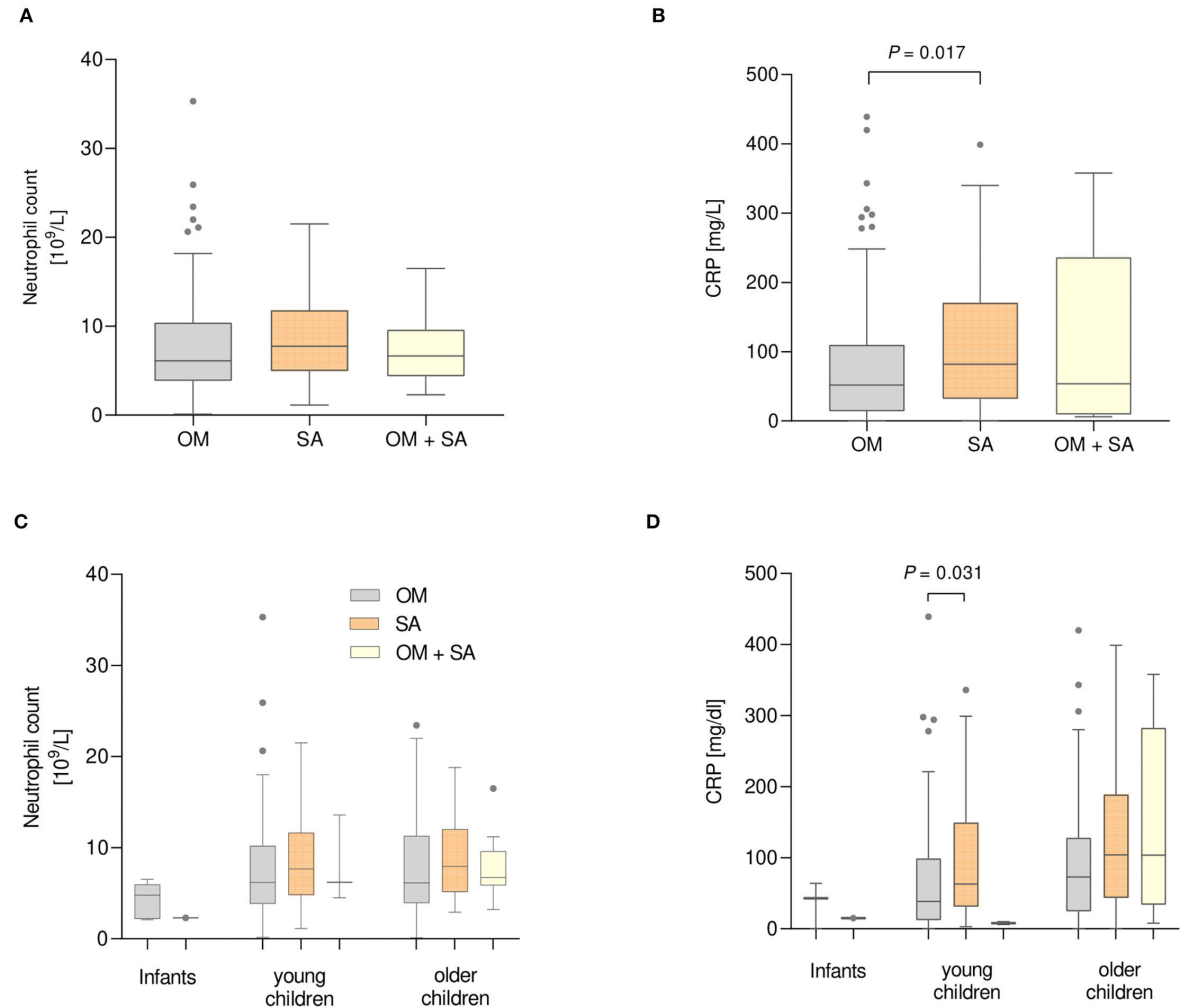
Neutrophil count **(A)** and C-reactive Protein (CRP) levels **(B)** measured in serum of patients with Bone and Joint infections. **(A)** *n* = 118 OM, 86 SA and 14 OM +SA patients. **(B)** *n* = 134 OM, 101 SA and 14 OM + SA patients. Neutrophil count **(C)** and CRP levels **(D)** of patients divided into three age groups. **(C)** *n* = 5 infants, 44 young and 69 older children with OM; 56 young and 30 older children with SA; 1 infant, 3 young and 10 older children with OM + SA. (D) *n* = 3 infants, 48 young and 83 older children with OM; 61 young and 40 older children with SA; and 1 infant, 3 young and 10 older children in the OM + SA group. Kruskal-Wallis test with Dunn's correction.

Neutrophils were not significantly different between the disease groups (Figures 5A,C). Patients with SA showed higher median CRP serum levels than patients with OM [82 mg/L vs. 52 mg/L, (*p* = 0.017)] (Figure 5B). Comparisons between the age groups revealed that young children with SA showed higher serum CRP levels than young children with OM [(63 mg/L vs. 38 mg/L, (*p* = 0.031)] (Figure 5D).

### Antibiotic Therapy

**Figure 6:**
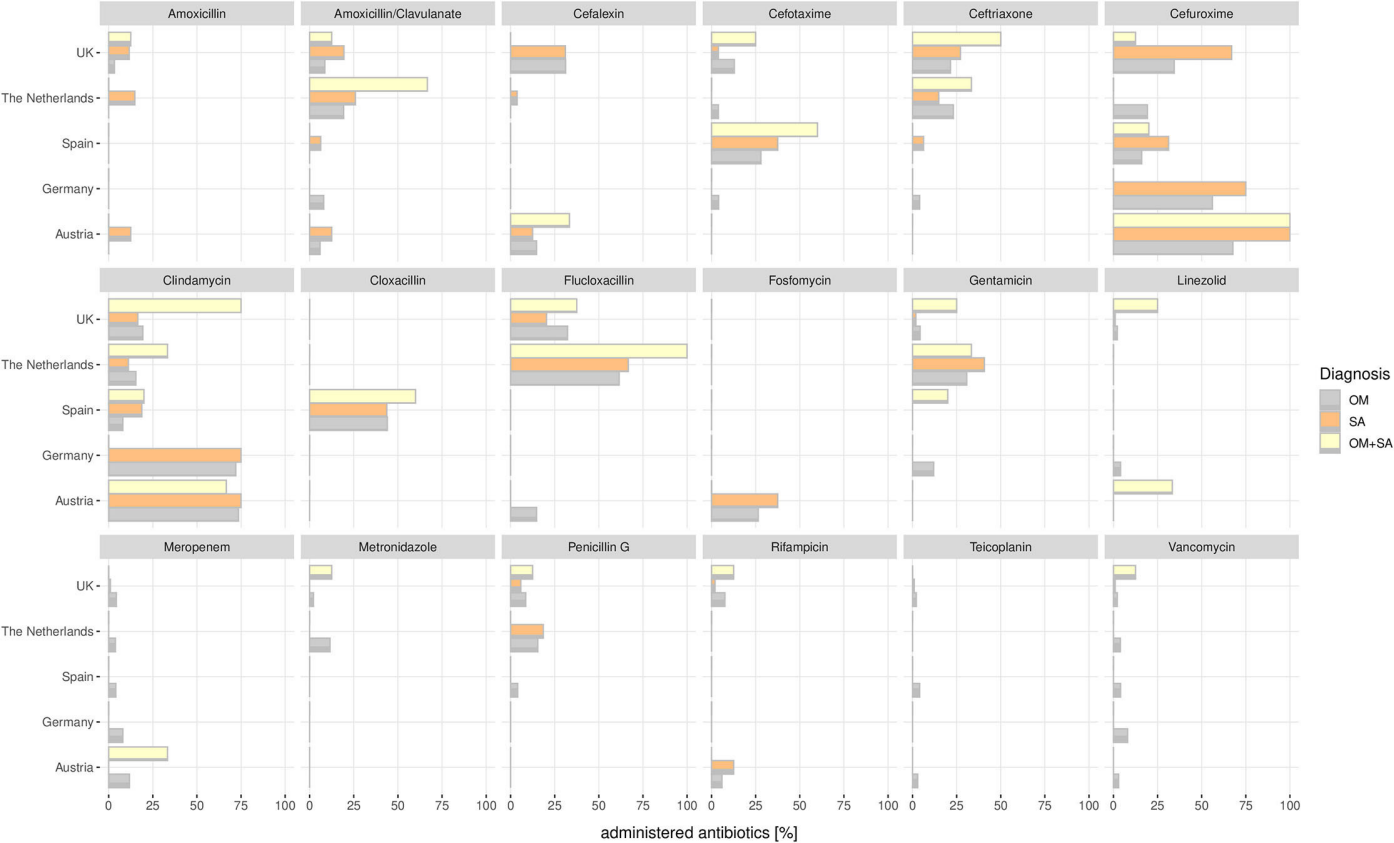
Percent of administered antibiotics calculated for each country in the consortium and each OAI are shown.

Cefuroxime [168/380 (44.2%)], clindamycin [109/380 (28.6%)], and flucloxacillin [96/380 (25.2%)] were most frequently prescribed among all patients with OAIs and all countries included in the consortium (Figures 1, 6). The main three substances were the same in patients with OM—cefuroxime [78/203 (38.4%)], clindamycin [67/203 (33%)], and flucloxacillin [51/203 (25.1%)]; in patients with SA—cefuroxime [58/158 (36,7%)], flucloxacillin [39/158 (24.6%)], and clindamycin [32/158 (20.3%)]; and in patients with both OM and SA—clindamycin [10/19 (52.6%)], flucloxacillin [6/19 (31.5%)], and cefuroxime [5/19 (26.3%)].

Comparison of antimicrobial differences between the countries revealed that the UK and the Netherlands showed higher administrations of flucloxacillin [UK, 54/204 (26.5%); the Netherlands, 37/57 (64.9%)] and ceftriaxone [UK, 52/204 (25.5%); The Netherlands, 11/57 (19.3%)]. In Austria, patients with OM and SA are mainly treated with clindamycin [25/34 (73.5%) vs. 6/8 (75%)] and cefuroxime [23/34 (67.6%) vs. 8/8 (100%)]. In Germany as well, patients with OM and SA are mainly treated with clindamycin [18/25 (72%) vs. 3/4 (75%)] and cefuroxime [14/25 (56%) vs. 3/4 (75%)].

## Discussion

We describe in this study that children suffering from POAIs have a substantial burden of disease. Median hospitalization duration in children suffering from OM was 10 days, while it was 7 days for children with SA, which is shorter than described in other studies (14, 24). More than half of patients received orthopedic operations: in children suffering from OM, operations were performed in 40% of cases and in SA in 75%. Whether to operate or treat conservatively is an ongoing debate, as well as the length of antibiotic therapy (25, 26). In our study cohort, intravenous antibiotic therapy was given for a median of 8 days, with a median of 11 days for patients with OM and 5 days for patients with SA. Administered antibiotics showed a large variety when compared per country but generally most used antibiotics were cefuroxim (44.2%), clindamycin (28.6%), and flucloxacillin (25.2%). Further patterns were identified, such as ceftriaxone rarely being used in Austria, Germany, or Spain in comparison to the Netherlands and the United Kingdom. Alternatively, in Austria and Germany, more than 70% of patients with OM and SA were treated with clindamycin.

According to the POPC Score 301 of 340 (88.5%) of patients with POAIs showed a good overall performance at discharge, 36 (10.5%) a mild overall disability, 3 (0.8%) a moderate, and 1 (0.2%) a severe overall disability. Patients with a combination of OM and SA showed more mild disabilities at discharge than the other patient groups. Only 5% of patients were diagnosed with a combination of OM and SA in our cohort. This is in the lower range compared to the literature, where incidences from 5.5 to 68% are reported, depending on the imaging used (27–29). Development of sequelae (or a trend thereof) associated with OM and SA is also reported in the literature (29, 30) and might be the result of diagnostic delay or undertreatment.

Albeit advances in diagnosis and treatment, pathogen detection is still challenging. Overall, no organism was found in more than one-third (35%) of patients, with negative results being highest in patients with OM (40%). This finding is substantiated by the literature, where 35% of negative pathogen detection rates in blood cultures or tissue samples were reported (31).

Blood culture was the primary detection method in patients with OM in our study, while in patients with SA causative pathogens were predominantly identified through blood culture as well as PCR from joint aspirates.

*Staphylococcus aureus* was the main microorganism identified, with higher detection rates in older children and in children with OM. One patient with MRSA was found in our cohort, which is in accordance with the European literature (5, 13, 32). Only 4% of *S. aureus* cases were reported as being PVL-positive, which is in contrast to recent studies reporting incidence rates of up to 18.6% in Europe (15).

Being part of the study protocol the question about the PVL-producing *S. aureus* was answered in 82.5% (113/137) of cases with “unknown”, in 13.1% (18/137) with “no” and in 4.4% (6/137) with “yes” in the database. This indicates that the virulence factor was not investigated in all patients of our cohort, but despite the small number, our cohort shows a detection of PVL in 25% (6/24) of all samples investigated for PVL-producing *S. aureus*. PVL is linked to CA-MRSA as well as to methicillin-susceptible *S*. *aureus* strains. (33). As infections assigned to *S. aureus* harboring this cytotoxin might be associated with higher disease severity (15, 16, 34), we recommend testing of PVL in each case of POAI caused by *S. aureus*. According to the European Society for Paediatric Infectious Diseases (ESPID) guidelines, adjunctive therapy with, e.g., clindamycin to inhibit toxin production is beneficial (19). All six of our cases with PVL-producing *S. aureus* had systemic infections, but only one needed PICU treatment.

Infections with GAS were predominantly found in patients with SA, 3–60 months of age, and resulted in amputations and skin grafts for three patients. Although this pathogen was reported to cause severe infections and sequelae mainly in young children (35) all three belonged to the older children group (median age, 117 months) as did patients with systemic infections due to GAS with PICU admission (median age, 90 months).

With advances in detection methods, *K. kingae* is increasingly reported in the etiology of bone and joint infections, most commonly detected in children with SA between 4 months and 5 years of age (30% to 94% of culture-positive OAIs) (6, 8, 36, 37). Children display mainly mild symptoms resulting in the presentation of none or very few criteria characteristic of OAIs and might therefore not be recognized (36). We also assume that there might be a higher number of missed *K. kingae* cases in our cohort, as not every microbiological laboratory routinely tested for this pathogen.

CRP is routinely used to monitor the response to therapy as well as the disease course and was increased in young and older children with SA in our cohort, which was also published by Paakkonen et al. (38). Due to the small number of patients with both OM and SA with recorded CRP (*n* = 14), we could not detect a difference in their levels among group comparisons.

Limitations of this study were incomplete data regarding blood counts, antibiotic treatment duration, data on overlapping intravenous/oral antibiotic administration and outcome, and a selection bias, since the original study design of EUCLIDS focused on severe bacterial infections due to *N. meningitidis, S. pneumoniae, S. aureus*, and GAS. Some centers also had a selection bias toward OM. Further, we assume that in some centers *K. kingae*-specific detection methods were not routinely performed. *K. kingae* rarely causes life-threatening infections (6), thus not necessarily meeting inclusion criteria for the EUCLIDS cohort. Therefore, the percentage of *K. kingae* is likely underestimated in our cohort. Furthermore, data on negative PCR testing were not routinely recorded.

## Conclusion

POAIs represent a substantial burden in European children, and further steps toward vaccination against *S. aureus* are urgently needed. Tests for the virulence factor PVL should be performed in patients where *S. aureus* is identified as causative organism. In addition, our findings suggest that children younger than 5 years suffering of SA should routinely be investigated for *K. kingae*, if possible by PCR. Our observations emphasize an urgent need for the development of updated microbiologic diagnostic guidelines for POAIs in European hospitals.

## Data Availability Statement

The data analyzed in this study is subject to the following licenses/restrictions: No restrictions, datasets can be accessed upon request. Requests to access these datasets should be directed to nina.schweintzger@medunigraz.at.

## Ethics Statement

Written informed consent was obtained from the minor(s)' legal guardian/next of kin for the publication of any potentially identifiable images or data included in this article.

## Author Contributions

AT and NS were equally involved in data processing and writing/editing of the manuscript. JH, EC, SP, ME, MF, RG, MC-L, IR-C, NB, P-MA, FS, SA, and FM-T acquired and processed samples/data in respective centers across europe and managed shipment of samples for further analysis. UB, UW, and KR contributed significantly to samples acquired for the austrian node. AG and GF provided microbiological data. AT, MSa, DK, and MSp were involved in patient/sample/data acquisition at Medical University Graz as well as storage/shipment of samples for further analysis. All authors helped in proof-reading, editing, reviewing, and approving the manuscript.

## Funding

EUCLIDS was funded by the European Union's Seventh Framework Programme (FP7; EC-GA No. 279185). The Austrian node received funding by the Department for Science and Research of the Styrian Federal Government (Austria), GZ: Abt.08-16.K-8/2012-20, and an ESPID grant 2011 for Endowed professorship for pediatric infectious diseases paying particular attention to meningococcal disease at the Department of General Pediatrics of the Medical University of Graz.

## Conflict of Interest

The authors declare that the research was conducted in the absence of any commercial or financial relationships that could be construed as a potential conflict of interest.

## Publisher's Note

All claims expressed in this article are solely those of the authors and do not necessarily represent those of their affiliated organizations, or those of the publisher, the editors and the reviewers. Any product that may be evaluated in this article, or claim that may be made by its manufacturer, is not guaranteed or endorsed by the publisher.
