# Supplementary material for: Osteoarticular Infections in Pediatric Hospitals in Europe: A Prospective Cohort Study From the EUCLIDS Consortium
Source: Front Pediatr. 2022 May 4;10:744182. doi: 10.3389/fped.2022.744182 (PMC9114665; doi:10.3389/fped.2022.744182)
Supplement: Supplementary file 1 [file Data_Sheet_1.PDF]

Supplemental Files

Suppl. Table 1: Primers used for PCR diagnostics (Microbiological laboratory Great Ormond Street Hospital (GOSH), London)

**Organism**	**Target gene**	**Assay sensitivity**	**Primer and probe sequences**
Staphylococcus aureus	coa	0,1-1 cfu/reaction	coa-forward: 5‘-GTAGATTGGGCAATTACATTTTGGAGG
			coa-reverse: 5‘-CGCATCTGCTTTGTTATCCCATGTA
			coa-probe: 5‘FAM-TAGGCGCATTAGCAGTTGCATC-BHQ1
Streptococcus pyogenes	csrR	0,1-1 cfu/reaction	GAS-forward: 5‘-TGGATGTGGTTGCAGGTTTAGAC
			GAS-reverse: 5‘-CGGGCAAGTAGTTCTTCAATGG
			GAS-probe: 5‘-JOE-CGGTGCAGACGACTATATTGTTAAACC-BHQ1
Streptococcus pneumoniae	lytA	0,1-1 cfu/reaction	lytA-forward: 5‘-ACGCAATCTAGCAGATGAAGC
			lytA-reverse: 5‘-TGTTTGGTTGGTTATTCGTGC
			lytA-probe: 5‘FAM-TTTGCCGAAAACGCTTGATACAGGG-BHQ1
Kingella kingae	Cpn60	Detection limit 300 cfu	KK(Cpn60)-1: 5’-GCTTTGGTTGGCGAATTGGC
			KK(Cpn60)-2-MOD: 5’-GGYGACAAGTAGCCRCGGTC
			KK(Cpn60)-probe: 5’-JOE-ACGAGCAAATCGCTCAAGTGGGCGC-BHQ1
