# Supplementary material for: Osteoarticular Infections in Pediatric Hospitals in Europe: A Prospective Cohort Study From the EUCLIDS Consortium
Source: Front Pediatr. 2022 May 4;10:744182. doi: 10.3389/fped.2022.744182 (PMC9114665; doi:10.3389/fped.2022.744182)
Supplement: Supplementary file 2 [file Data_Sheet_2.PDF]

Suppl. Table 2. **The Pediatric Overall Performance Category (POPC) scale.**

Score	Category	Description
**1**	Good overall performance	Normal; at age-appropriate level; school-age child attending regular school classroom.<br>Healthy, alert, and capable of normal activities of daily life.
**2**	Mild overall disability	Conscious; alert, and able to interact at age-appropriate level; school-age child attending regular school classroom but grade perhaps not appropriate for age; possibility of mild neurologic deficit.<br>Possibility of minor physical problem that is still compatible with normal life; conscious and able to function independently.
**3**	Moderate overall disability	Conscious; sufficient cerebral function for age-appropriate independent activities of daily life; school-age child attending special education classroom and/or learning deficit present<br>Possibility of moderate disability from noncerebral systems dysfunction alone or with cerebral system dysfunction; conscious and performs independent activities of daily life but is disabled for competitive performance in school.
**4**	Severe overall disability	Conscious; dependent on others for daily support because of impaired brain function.<br>Possibility of severe disability from noncerebral systems dysfunction alone or with cerebral system dysfunction; conscious but dependent on others for activities of daily living support.
**5**	Coma or vegetative state	Any degree of coma without the presence of all brain death criteria; unawareness, even if awake in appearance, without interaction with environment; cerebral unresponsiveness and no evidence of cortex function (not aroused by verbal stimuli); possibility of some reflexive response, spontaneous eye-opening, and sleep-wake cycles.
**6**	Brain death	Apnea, areflexia, and/or electroencephalographic silence.

Suppl. file A. Clinical definitions:

Osteomyelitis was defined as an inflammation or an infection in the bone marrow or surrounding bone and classified according to duration of symptoms in acute (< 14 days), subacute (14 days to a month) or chronic (> 1 month). Diagnosis was based on following criteria, of which at least two must be positive: (1) Presence of localized pain/tenderness and other typical features of osteomyelitis such as warmth and/or swelling of the affected region. AND/OR (2) image findings consistent with osteomyelitis (typical MRI findings and/or positive bone scan) AND/OR (3) bacteriologic evidence of infection (positive blood and/or bone culture) AND/OR (4) histopathological finding consistent with osteomyelitis (intraoperative specimen)

Septic arthritis was diagnosed when a microorganism was isolated from the blood in a child with clinical arthritis; or by aspiration of synovial fluid and/or purulent fluid from the joint, in which the white blood cell count was > (WBC) 50 000/mm3.

MRI findings consistent with acute/subacute/chronic osteomyelitis: On unenhanced images, osteomyelitis was characterized by focally decreased marrow signal intensity on T1-weighted images AND focally increased marrow signal intensity on fluid-sensitive images (fat-suppressed T2-weighted and STIR sequences). OR following contrast administration, osteomyelitis was described as focal abnormal bone marrow enhancement on fat-suppressed T1-weighted images. Complications of osteomyelitis include abscesses - intraosseous, subperiosteal, and soft-tissue abscesses are defined as well circumscribed areas of focally decreased signal intensity on T1-weighted images with increased signal intensity equal to that of fluid on fluid-sensitive sequences and/or rim enhancement on contrast-enhanced fat-suppressed T1-weighted images.

Subacute osteomyelitis can manifest as Brodie abscess characterized by a central abscess cavity filled with fluid, an inner ring of enhancing high signal intensity granulation tissue on T1-weighted sequences, an outer ring of very low signal intensity sclerosis, and a peripheral halo of edema.

In chronic osteomyelitis, imaging might reveal an involucrum (thick periosteal new bone), sequestrum (necrotic bone fragment), or cloaca (draining tract through a defect in the cortex and involucrum).

Bone scan consistent with osteomyelitis (Technetium-99m bone scan): The most definitive phase is the delayed phase: There is no osteomyelitis without abnormal radionuclide uptake on the images obtained during the delayed phase, even if there is increased activity on blood flow or blood pool images.

AND/OR

The hallmark feature of osteomyelitis at 99mTc scintigraphy is increased activity in all three phases (1. Angiographic or blood flow phase, 2. Blood pool or tissue phase and 3. Delayed phase).

Histopathological finding consistent with acute/subacute/chronic osteomyelitis include presence of inflammatory cells (in acute osteomyelitis: predominantly polymorphonuclear leucocytes, in chronic osteomyelitis: mononuclear cells including plasma cells and macrophage/monocyte cells) AND/OR destruction/necrosis of bone (necrotic marrow and bone, osteoclastic activity) AND/OR granulation tissue (hemorrhage, polymorphonuclear leucocytes, lymphocytes, and macrophages). In implant-associated infections, tissue specimens obtained for histopathology either by biopsy or during surgery as frozen section are important because the presence of neutrophils in significant amounts is indicative of infection. More than five neutrophils per high-power field indicate infection, with sensitivity of 43-84% and specificity of 93-97%. These infections will be considered as “community acquired” depending on the onset of symptoms after implantation: after 24 months it is considered to be a community acquired infection.

Discitis/Spondylodiscitis is an inflammatory process involving the intervertebral disks and the endplates of the vertebral bodies, and associated with characteristic clinical and radiologic findings.
Mastoiditis is a suppurative infection of the mastoid air cells, and the most common suppurative complication of acute otitis media. In acute mastoiditis, symptoms are of less than 1 month´s duration. There is a lack of consensus regarding the criteria and strategies for diagnosing acute mastoiditis in the pediatric population. The diagnosis is usually made clinically [fever, otalgia, post-auricular erythema, tenderness, swelling, fluctuance or mass, displacement of the auricle (down and out: children <2 years); up and out in children ≥2 years] without need for imaging studies. At imaging CT and MRI show haziness or destruction of the mastoid outline; and loss of or decrease in the sharpness of the bony septa that define the mastoid air cells. Positive culture or gram stain of a specimen obtained from the middle ear either by tympanocentesis through an intact eardrum or by aspiration through a tympanostomy tube or perforation is warranted.

EUCLIDS Consortium Author list:
Imperial College partner (UK)
Members of the EUCLIDS Consortium at Imperial College London (UK)
Principal and co-investigators
Michael Levin (grant application, EUCLIDS Coordinator)
Dr. Lachlan Coin (bioinformatics)
Stuart Gormley (clinical coordination)
Shea Hamilton (proteomics)
Jethro Herberg (grant application, PI)
Bernardo Hourmat (project management)
Clive Hoggart (statistical genomics)
Myrsini Kaforou (bioinformatics)
Vanessa Sancho-Shimizu (genetics)
Victoria Wright (grant application, scientific coordination)
Consortium members at Imperial College
Amina Abdulla
Paul Agapow
Maeve Bartlett
Evangelos Bellos
Hariklia Eleftherohorinou
Rachel Galassini
David Inwald
Meg Mashbat
Stefanie Menikou
Sobia Mustafa
Simon Nadel
Rahmeen Rahman
Clare Thakker

EUCLIDS UK Clinical Network

Poole Hospital NHS Foundation Trust, Poole: Dr S Bokhandi (PI), Sue Power, Heather Barham
Cambridge University Hospitals NHS Trust, Cambridge: Dr N Pathan (PI), Jenna Ridout, Deborah White, Sarah Thurston

University Hospital Southampton, Southampton: Prof S Faust (PI), Dr S Patel (co-investigator), Jenni McCorkell.

Nottingham University Hospital NHS Trust: Dr P Davies (PI), Lindsey Crate, Helen Navarra, Stephanie Carter

University Hospitals of Leicester NHS Trust, Leicester: Dr R Ramaiah (PI), Rekha Patel

Portsmouth Hospitals NHS Trust, London: Dr Catherine Tuffrey (PI), Andrew Gribbin, Sharon McCready

Great Ormond Street Hospital, London: Dr Mark Peters (PI), Katie Hardy, Fran Standing, Lauren O'Neill, Eugenia Abelake

King's College Hospital NHS Foundation Trust, London; Dr Akash Deep (PI), Eniola Nsirim

Oxford University Hospitals NHS Foundation Trust, Oxford Prof A Pollard (PI), Louise Willis, Zoe Young

Kettering General Hospital NHS Foundation Trust, Kettering: Dr C Royad (PI), Sonia White

Central Manchester NHS Trust, Manchester: Dr PM Fortune (PI), Phil Hudnott

Austrian partner

PI: Werner Zenz[1]
Co-Investigators:
Daniela S. Kohlfürst[1], Alexander Binder[1], Nina A. Schweintzger[1], Manfred G. Sagmeister[1]

[1]University Clinic of Pediatrics and Adolescent Medicine, Department of General Pediatrics, Medical University Graz, Austria

Austrian network, participating centers in Austria, Germany, Italy, Serbia, Lithuania, patient recruitment (in alphabetical order):
Hinrich Baumgart[1], Markus Baumgartner[2], Uta Behrends[3], Ariane Biebl[4], Robert Birnbacher[5], Jan-Gerd Blanke[6], Carsten Boelke[7], Kai Breuling[3], Jürgen Brunner[8], Maria Buller[9], Peter Dahlem[10], Beate Dietrich[11], Ernst Eber[12], Johannes Elias[13], Josef Emhofer[2], Rosa Etschmaier[14], Sebastian Farr[15], Ylenia Girtler[16], Irina Grigorow[17], Konrad Heimann[18], Ulrike Ihm[19], Zdenek Jaros[20], Hermann Kalhoff[21], Wilhelm Kaulfersch[22], Christoph Kemen[23], Nina Klocker[24], Bernhard Köster[25], Benno Kohlmaier[26], Eleni Komini[27], Lydia Kramer[3], Antje Neubert[28], Daniel Ortner[29], Lydia Pescollderungg[16], Klaus Pfurtscheller[30], Karl Reiter[31], Goran Ristic[32], Siegfried Rödl[30], Andrea Sellner[26], Astrid Sonnleitner[26], Matthias Sperl[33], Wolfgang Stelzl[34], Holger Till[1], Andreas Trobisch[26], Anne Vierzig[35], Ulrich Vogel[12], Christina Weingarten[36], Stefanie Welke[37], Andreas Wimmer[38], Uwe Wintergerst[39], Daniel Wüller[40], Andrew Zaunschirm[41], Ieva Ziuraite[42], Veslava Žukovskaja[42]

[1]Department of Pediatric and Adolescence Surgery, Division of General Pediatric Surgery, Medical University Graz, Austria
[2]Department of Pediatrics, General Hospital of Steyr, Austria
[3]Department of Pediatrics/Department of Pediatric Surgery, Technische Universität München (TUM), Munich, Germany
[4]Department of Pediatrics, Kepler University Clinic, Medical Faculty of the Johannes Kepler University, Linz, Austria
[5]Department of Pediatrics and Adolescent Medicine LKH Villach, Austria
[6]Department of Pediatrics and Adolescent Medicine and Neonatology, Hospital Ludmillenstift, Meppen, Germany
[7]Hospital for Children's and Youth Medicine, Oberschwabenklinik, Ravensburg, Germany
[8]Department of Pediatrics, Medical University Innsbruck, Austria
[9]Clinic for Pediatrics and Adolescents Medicine, Sana Hanse-Klinikum Wismar, Germany
[10]Departement of Pediatrics, Medical Center Coburg, Germany
[11]University Medicine Rostock, Department of Pediatrics (UKJ), Rostock, Germany
[12]Department of Pulmonology, Medical University Graz, Austria

[13]Institute for Hygiene and Microbiology, University of Würzburg, Germany
[14]Clinical Institute of Medical and Chemical Laboratory Diagnostics, Medical University Graz, Austria
[15]Department of Pediatric Orthopedics and Adult Foot and Ankle Surgery, Orthopedic Hospital Speising, Vienna, Austria
[16]Department of Pediatrics, Regional Hospital Bolzano, Italy
[17]Department of Pediatrics and Adolescent Medicine, General Hospital Hochsteiermark/Leoben, Austria
[18]Department of Neonatology and Pediatric Intensive Care, Children's University Hospital, RWTH Aachen, Germany
[19]Paediatric Intensive Care Unit, Department of Pediatric Surgery, Donauspital Vienna, Austria
[20]Department of Pediatrics, General Public Hospital, Zwettl, Austria
[21]Pediatric Clinic Dortmund, Germany
[22]Department of Pediatrics and Adolescent Medicine, Klinikum Klagenfurt am Wörthersee, Klagenfurt, Austria
[23]Catholic Children's Hospital Wilhelmstift, Department of Pediatrics, Hamburg, Germany
[24]Department of Pediatrics, Krankenhaus Dornbirn, Austria
[25]Children’s Hospital Luedenscheid, Maerkische Kliniken, Luedenscheid, Germany
[26]Department of General Pediatrics, Medical University Graz, Austria
[27]Department of Pediatrics, Schwarzwald-Baar-Hospital, Villingen-Schwenningen, Germany
[28]Department of Pediatrics and Adolescents Medicine, University Hospital Erlangen, Germany
[29]Department of Pediatrics and Adolescent Medicine, Medical University of Salzburg, Austria
[30]Pediatric Intensive Care Unit, Medical University Graz, Austria
[31]Dr. von Hauner Children's Hospital, Ludwig-Maximilians- Universitaet, Munich, Germany
[32]Mother and Child Health Care Institute of Serbia, Belgrade, Serbia
[33]Department of Pediatric and Adolescence Surgery, Division of Pediatric Orthopedics, Medical University Graz, Austria
[34]Department of Pediatrics, Academic Teaching Hospital, Landeskrankenhaus Feldkirch, Austria
[35]University Children’s Hospital, University of Cologne, Germany
[36]Department of Pediatrics and Adolescent Medicine Wilheminenspital, Vienna, Austria
[37]Department of Pediatric Surgery, Municipal Hospital Karlsruhe, Germany
[38]Hospital of the Sisters of Mercy Ried, Department of Pediatrics and Adolescent Medicine, Ried, Austria
[39]Hospital St. Josef, Braunau, Austria
[40]Christophorus Kliniken Coesfeld Clinic for Pediatrics, Coesfeld, Germany
[41]Department of Pediatrics, University Hospital Krems, Karl Landsteiner University of Health Sciences, Krems, Austria
[42]Children‘s Hospital, Affiliate of Vilnius University Hospital Santariskiu Klinikos, Lithuania

Microbiology (pathogen isolates):
Claudia Mikula[1]
[1]Austrian Agency for Health and Food Safety, Institute for Medical Microbiology and Hygiene Graz, Austria
Gebhard Feierl[2]
[2]Institute of Hygiene, Microbiology and Environmental Medicine, Medical University Graz, Austria

SERGAS Partner (Spain)
Principal Investigators
Federico Martinón-Torres[1]
Antonio Salas[1,2]

GENVIP RESEARCH GROUP (in alphabetical order):
Fernando Álvez González[1], Ruth Barral-Arca[1,2], Miriam Cebey-López[1], María José Curras-Tuala[1,2], Natalia García[1], Luisa García Vicente[1], Alberto Gómez-Carballa[1,2], Jose Gómez Rial[1], Andrea Grela Beiroa[1], Antonio Justicia Grande[1], Pilar Leboráns Iglesias[1], Alba Elena Martínez Santos[1], Federico Martinón-Torres[1], Nazareth Martinón-Torres[1], José María Martinón Sánchez[1], Beatriz Morillo Gutiérrez[1], Belén Mosquera Pérez[1], Pablo Obando Pacheco[1], Jacobo Pardo-Seco[1,2], Sara Pischedda[1,2], Irene Rivero Calle[1], Carmen Rodríguez-Tenreiro[1], Lorenzo Redondo-Collazo[1], Antonio Salas Ellacuriaga[1,2], Sonia Serén Fernández[1], María del Sol Porto Silva[1], Ana Vega[1,3,] Lucía Vilanova Trillo[1].
[1] Translational Pediatrics and Infectious Diseases, Pediatrics Department, Hospital Clínico Universitario de Santiago, Santiago de Compostela, Spain, and GENVIP Research Group (www.genvip.org), Instituto de Investigación Sanitaria de Santiago, Galicia, Spain.
[2] Unidade de Xenética, Departamento de Anatomía Patolóxica e Ciencias Forenses, Instituto de Ciencias Forenses, Facultade de Medicina, Universidade de Santiago de Compostela, and GenPop Research Group, Instituto de Investigaciones Sanitarias (IDIS), Hospital Clínico Universitario de Santiago, Galicia, Spain
[3] Fundación Pública Galega de Medicina Xenómica, Servizo Galego de Saúde (SERGAS), Instituto de Investigaciones Sanitarias (IDIS), and Grupo de Medicina Xenómica, Centro de Investigación Biomédica en Red de Enfermedades Raras (CIBERER), Universidade de Santiago de Compostela (USC), Santiago de Compostela, Spain

EUCLIDS SPANISH CLINICAL NETWORK:
Susana Beatriz Reyes[1], María Cruz León León[1], Álvaro Navarro Mingorance[1], Xavier Gabaldó Barrios[1], Eider Oñate Vergara[2], Andrés Concha Torre[3], Ana Vivanco[3], Reyes Fernández[3], Francisco Giménez Sánchez[4], Miguel Sánchez Forte[4], Pablo Rojo[5], J.Ruiz Contreras[5], Alba Palacios[5], Cristina Epalza Ibarrondo[5], Elizabeth Fernandez Cooke[5], Marisa Navarro[6], Cristina Álvarez Álvarez[6], María José Lozano[6], Eduardo Carreras[7], Sonia Brió Sanagustín[7], Olaf Neth[8], Mª del Carmen Martínez Padilla[9], Luis Manuel Prieto Tato[10], Sara Guillén[10], Laura Fernández Silveira[11], David Moreno[12].
[1] Hospital Clínico Universitario Virgen de la Arrixaca; Murcia, Spain.
[2] Hospital de Donostia; San Sebastián, Spain.
[3] Hospital Universitario Central de Asturias; Asturias, Spain.
[4] Complejo Hospitalario Torrecárdenas; Almería, Spain.
[5] Hospital Universitario 12 de Octubre; Madrid, Spain.
[6] Hospital General Universitario Gregorio Marañón; Madrid, Spain.
[7] Hospital de la Santa Creu i Sant Pau; Barcelona, Spain.
[8] Hospital Universitario Virgen del Rocío; Sevilla, Spain.
[9] Complejo Hospitalario de Jaén; Jaén, Spain.
[10] Hospital Universitario de Getafe; Madrid, Spain.
[11] Hospital Universitario y Politécnico de La Fe; Valencia, Spain.
[12] Hospital Regional Universitario Carlos Haya; Málaga, Spain.

FUNDING:
Instituto de Salud Carlos III (Proyecto de Investigación en Salud, Acción Estratégica en Salud: proyecto GePEM PI16/01478) (A.S.); Consellería de Sanidade, Xunta de Galicia (RHI07/2-intensificación actividad investigadora, PS09749 and 10PXIB918184PR), Instituto de Salud Carlos III (Intensificación de la actividad investigadora 2007–2016), Convenio de colaboración de investigación (Wyeth España-Fundación IDICHUS 2007–2011), Convenio de colaboración de investigación (Novartis España-Fundación IDICHUS 2010–2011), Fondo de Investigación Sanitaria (FIS; PI070069/PI1000540) del plan nacional de I + D + I and 'fondos FEDER' (F.M.T.).

Members of the Pediatric Dutch Bacterial Infection Genetics (PeD-BIG) network (the Netherlands)

Steering committee:
Coordination: R. de Groot [1], A.M. Tutu van Furth [2], M. van der Flier [1]
Coordination Intensive Care: N.P. Boeddha [3], G.J.A. Driessen [3], M. Emonts [3], J.A. Hazelzet [3]
Other members: T.W. Kuijpers [5], D. Pajkrt [5], E.A.M. Sanders [4] , D. van de Beek [6], A. van der Ende[6]
Trial coordinator: H.L.A. Philipsen [1]

Local investigators (in alphabetical order)
A.O.A. Adeel [7], M.A. Breukels [8], D.M.C. Brinkman [9], C.C.M.M. de Korte [10], E. de Vries [11], W.J. de Waal [12], R. Dekkers [13], A. Dings-Lammertink [14], R.A. Doedens [15], A.E. Donker [16], M. Dousma[17], T.E. Faber [18], G.P.J.M. Gerrits[19], J.A.M. Gerver [20], J. Heidema [21], J. Homan-van der Veen [22], M.A.M. Jacobs [23], N.J.G. Jansen [4], P. Kawczynski [24], K. Klucovska [25], M.C.J. Kneyber [26], Y. Koopman-Keemink [27], V.J. Langenhorst [28], J. Leusink [29], B.F. Loza [30], I.T. Merth [31], C.J. Miedema [32], C. Neeleman [1], J.G. Noordzij [33], C.C. Obihara [34], A.L.T. van Overbeek – van Gils [35], G.H. Poortman [36],S.T. Potgieter [37], J. Potjewijd [38], P.P.R. Rosias [39], T. Sprong [19], G.W. ten Tussher [40], B.J. Thio [41], G.A. Tramper-Stranders [42], M. van Deuren [1], H. van der Meer [2], A.J.M. van Kuppevelt [43], A.M. van Wermeskerken [44], W.A. Verwijs [45], T.F.W. Wolfs [4].

1. Radboud University Medical Center – Amalia Children's Hospital, Nijmegen, The Netherlands
2. Vrije Universiteit University Medical Center, Amsterdam, The Netherlands
3. Erasmus Medical Center – Sophia Children's Hospital, Rotterdam, The Netherlands
4. University Medical Center Utrecht – Wilhelmina Children's Hospital, Utrecht, The Netherlands
5. Academic Medical Center – Emma Children's Hospital, University of Amsterdam, Amsterdam, The Netherlands
6. Academic Medical Center, University of Amsterdam, Amsterdam, The Netherlands
7. Kennemer Gasthuis, Haarlem, The Netherlands
8. Elkerliek Hospital, Helmond, The Netherlands
9. Alrijne Hospital, Leiderdorp, The Netherlands

10. Beatrix Hospital, Gorinchem, The Netherlands
11. Jeroen Bosch Hospital, ‘s-Hertogenbosch, The Netherlands
12. Diakonessenhuis, Utrecht, The Netherlands
13. Maasziekenhuis Pantein, Boxmeer, The Netherlands
14. Gelre Hospitals, Zutphen, The Netherlands
15. Martini Hospital, Groningen, The Netherlands
16. Maxima Medical Center, Veldhoven, The Netherlands
17. Gemini Hospital, Den Helder, The Netherlands
18. Medical Center Leeuwarden, Leeuwarden, The Netherlands
19. Canisius-Wilhelmina Hospital, Nijmegen, The Netherlands
20. Rode Kruis Hospital, Beverwijk, The Netherlands
21. St. Antonius Hospital, Nieuwegein, The Netherlands
22. Deventer Hospital, Deventer, The Netherlands
23. Slingeland Hospital, Doetinchem, The Netherlands
24. Refaja Hospital, Stadskanaal, The Netherlands
25. Bethesda Hospital, Hoogeveen, The Netherlands
26. University Medical Center Groningen, Beatrix Children’s hospital, Groningen, The Netherlands
27. Haga Hospital – Juliana Children’s Hospital, Den Haag, The Netherlands
28. Isala Hospital, Zwolle, The Netherlands
29. Bernhoven Hospital, Uden, The Netherlands
30. VieCuri Medical Center, Venlo, The Netherlands
31. Ziekenhuisgroep Twente, Almelo-Hengelo, The Netherlands
32. Catharina Hospital, Eindhoven, The Netherlands
33. Reinier de Graaf Gasthuis, Delft, The Netherlands
34. ETZ Elisabeth, Tilburg, The Netherlands
35. Scheper Hospital, Emmen, The Netherlands
36. St. Jansdal Hospital, Hardewijk, The Netherlands
37. Laurentius Hospital, Roermond, The Netherlands
38. Isala Diaconessenhuis, Meppel, The Netherlands
39. Zuyderland Medical Center, Sittard-Geleen, The Netherlands
40. Westfriesgasthuis, Hoorn, The Netherlands
41. Medisch Spectrum Twente, Enschede, The Netherlands
42. St. Franciscus Gasthuis, Rotterdam, The Netherlands
43. Streekziekenhuis Koningin Beatrix, Winterswijk, The Netherlands
44. Flevo Hospital, Almere, The Netherlands
45. Zuwe Hofpoort Hospital, Woerden, The Netherlands

Micropathology Ltd

Colin G Fink[1,2], Elli Pinnock[1]

[1]Micropathology Ltd Research and Diagnosis
[2]University of Warwick
Newcastle partner

Principle Investigator
Marieke Emonts[1,2, 3]

Co-Investigator
Rachel Agbeko[1,3,4]

[1] Translational and Clinical Research Institute, Newcastle University, Newcastle upon Tyne, United Kingdom

[2] Pediatric Infectious Diseases and Immunology Department, Newcastle upon Tyne Hospitals Foundation Trust, Great North Children's Hospital, Newcastle upon Tyne, United Kingdom
[3] NIHR Newcastle Biomedical Research Centre based at Newcastle upon Tyne Hospitals NHS Trust and Newcastle University, Westgate Rd, Newcastle upon Tyne 32 NE4 5PL, United Kingdom
[4] Pediatric Intensive Care Unit, Newcastle upon Tyne Hospitals Foundation Trust, Great North Children's Hospital, Newcastle upon Tyne, United Kingdom

Acknowledgement
The Research was supported by the National Institute for Health Research Newcastle Biomedical Research Centre based at Newcastle Hospitals NHS Foundation Trust and Newcastle University. The views expressed are those of the author(s) and not necessarily those of the NHS, the NIHR or the Department of Health.
Liverpool Partner

Principal Investigators
Enitan Carrol[1,2]
Stéphane Paulus [1,2]

ALDER HEY SERIOUS PAEDIATRIC INFECTION RESEARCH GROUP (ASPIRE) (in alphabetical order):
Hannah Frederick[3], Rebecca Jennings[3] , Joanne Johnston[3], Rhian Kenwright[3]

[1] Department of Clinical Infection, Microbiology and Immunology, University of Liverpool Institute of Infection, Veterinary and Ecological Sciences , Liverpool, England
[2] Alder Hey Children's Hospital, Department of Infectious Diseases, Eaton Road, Liverpool, L12 2AP
[3] Alder Hey Children's Hospital, Clinical Research Business Unit, Eaton Road, Liverpool, L12 2AP

Microbiological laboratory Great Ormond Street Hospital (GOSH), London
Gambia partner
-
Suzanne Anderson: Principal Investigator and West African study oversight:
Fatou Secka: Clinical research fellow and study co-ordinator

Additional Gambia site team (consortium members):
Kalifa Bojang: co-PI
Isatou Sarr: Senior laboratory technician
Ngane Kebbeh: Junior laboratory technician
Gibbi Sey: lead research nurse Medical Research Council Clinic

Momodou Saidykhan: lead research nurse Edward Francis Small Teaching Hospital
Fatoumatta Cole: Data manager
Gilleh Thomas: Data manager
Martin Antonio: Local collaborator

Medical Research Council Unit Gambia
PO Box 273
Banjul
The Gambia

Swiss Pediatric Sepsis Study

***Steering Committee**: Luregn J Schlapbach, MD, FCICM [1,2,3], Philipp Agyeman, MD [1], Christoph Aebi, MD [1], Christoph Berger, MD [1]*
Luregn J Schlapbach, MD, FCICM [1,2,3], Philipp Agyeman, MD [1], Christoph Aebi, MD [1], Eric Giannoni, MD [4,5], Martin Stocker, MD [6], Klara M Posfay-Barbe, MD [7], Ulrich Heininger, MD [8], Sara Bernhard-Stirnemann, MD [9], Anita Niederer-Loher, MD [10], Christian Kahlert, MD [10], Paul Hasters, MD [11], Christa Relly, MD [12], Walter Baer, MD [13], Christoph Berger, MD [12] **for the Swiss Pediatric Sepsis Study**

1. Department of Pediatrics, Inselspital, Bern University Hospital, University of Bern, Switzerland
2. Pediatric Critical Care Research Group, Mater Research Institute, University of Queensland, Brisbane, Australia
3. Pediatric Intensive Care Unit, Lady Cilento Children's Hospital, Children's Health Queensland, Brisbane, Australia
4. Service of Neonatology, Lausanne University Hospital, Lausanne, Switzerland
5. Infectious Diseases Service, Lausanne University Hospital, Lausanne, Switzerland
6. Department of Pediatrics, Children's Hospital Lucerne, Lucerne, Switzerland
7. Pediatric Infectious Diseases Unit, Children's Hospital of Geneva, University Hospitals of Geneva, Geneva, Switzerland
8. Infectious Diseases and Vaccinology, University of Basel Children's Hospital, Basel, Switzerland
9. Children's Hospital Aarau, Aarau, Switzerland
10. Division of Infectious Diseases and Hospital Epidemiology, Children's Hospital of Eastern Switzerland St. Gallen, St. Gallen, Switzerland
11. Department of Neonatology, University Hospital Zurich, Zurich, Switzerland
12. Division of Infectious Diseases and Hospital Epidemiology, and Children's Research Center, University Children's Hospital Zurich, Switzerland
13. Children's Hospital Chur, Chur, Switzerland

Funding
This study was funded by grants from the Swiss National Science Foundation (342730_153158/1), the Swiss Society of Intensive Care, the Bangerter Foundation, the Vinetum and Borer Foundation, and the Foundation for the Health of Children and Adolescents.
